# An evolutionary model of personality traits related to cooperative behavior using a large language model

**DOI:** 10.1038/s41598-024-55903-y

**Published:** 2024-03-19

**Authors:** Reiji Suzuki, Takaya Arita

**Affiliations:** Graduate School of Informatics, Nagoya University, Furo-cho, Chikusa-ku, Nagoya, 464-8601 Japan

**Keywords:** Cooperation, Evolution, Prisoner’s dilemma, Large language model, Personality trait, Artificial life, Computer science, Computational models, Cultural evolution, Evolutionary theory, Computer science, Computational models, Cultural evolution, Evolutionary theory

## Abstract

This study aims to demonstrate that Large Language Models (LLMs) can empower research on the evolution of human behavior, based on evolutionary game theory, by using an evolutionary model positing that instructing LLMs with high-level psychological and cognitive character descriptions enables the simulation of human behavior choices in game-theoretical scenarios. As a first step towards this objective, this paper proposes an evolutionary model of personality traits related to cooperative behavior using a large language model. In the model, linguistic descriptions of personality traits related to cooperative behavior are used as genes. The deterministic strategies extracted from LLM that make behavioral decisions based on these personality traits are used as behavioral traits. The population is evolved according to selection based on average payoff and mutation of genes by asking LLM to slightly modify the parent gene toward cooperative or selfish. Through experiments and analyses, we clarify that such a model can indeed exhibit evolution of cooperative behavior based on the diverse and higher-order representation of personality traits. We also observed repeated intrusion of cooperative and selfish personality traits through changes in the expression of personality traits. The words that emerged in the evolved genes reflected the behavioral tendencies of their associated personalities in terms of semantics, thereby influencing individual behavior and, consequently, the evolutionary dynamics.

## Introduction

Large Language Models (LLMs), such as ChatGPT, are rapidly transforming human interactions with AI and raising questions about the nature of human intelligence and consciousness^1^. It is essential to understand the interactions between artificial individuals based on LLMs^2^ and the societies in which humans and artificial individuals coexist.

Modeling approaches to the evolution of social populations have primarily been discussed within the framework of evolutionary game theory^3,4^, using mathematical and computational methods such as replicator dynamics and agent-based models. The evolution of behavioral strategies in the Prisoner’s Dilemma as an abstraction of social conflict is a seminal example. It has provided general insights into the evolution of cooperation in biological organisms and human society^5,6^.

Conventional models of the evolution of cooperative behavior have typically described specific actions in particular situations as direct representation of individual genes. However, such behaviors often stem from higher-order psychological or cognitive traits, including intentions, personality, and preferences. In psychology, the widely accepted “Big Five” model categorizes personality traits into five dimensions: Openness to Experience, Conscientiousness, Extraversion, Agreeableness and Neuroticism^7^. However, translating these traits into specific behaviors in diverse social contexts remains challenging, especially in mathematical and computational models.

This study aims to demonstrate that LLMs can empower research on the evolution of human behavior, based on evolutionary game theory, by using an evolutionary model positing that instructing LLMs with high-level psychological and cognitive character descriptions enables the simulation of human behavior choices in game-theoretical scenarios. As a first step towards this objective, this paper proposes an evolutionary model of personality traits related to cooperative behavior using a large language model. We apply the capability of LLM to output behavioral strategies in response to linguistic descriptions of personality. Phelps and Russell examined how GPT-3.5 operationalizes natural language descriptions of motivations, including competitiveness and altruism, etc., in social dilemmas^8^. Using prompts that described varying attitudes, they showed that LLMs can adequately interpret and demonstrate these traits in behavior, though with some limitations. We use natural language to represent personality trait genes in the model, which can be translated into behavioral traits by using LLMs.

We evolve such genes through a mutation method also employing the LLM. Meyerson et al. recently used LLMs for crossovers in evolutionary computation, by inputting several patterns as parents to LLM to generate their offspring patterns^9^. They showed evolution of diverse patterns, including binary strings, sentences, and Python code. We adopt similar concept but with a simpler method to mutate genes represented as natural language descriptions to evolve population of personality trait genes.

Furthermore, this level of personality description and generation of their behavioral traits enables the model to deal with evolution not only in specific game-theoretical situations but also in other game-theoretical contexts, as well as in any context that can be described linguistically.

This paper addresses the following research questions to clarify the significance of the proposed model: (1) How can behavioral traits generated from genes represented in natural language using the LLM-based method reflect genes' cooperative tendency and consistency? (2) Can the proposed model exhibit the complex evolutionary dynamics of the emergence of personality traits for cooperative behavior, and if so, how do these dynamics play out in a typical trial? (3) What are the statistical properties and the dynamics of the evolution process in the proposed model, and how do these compare with those of a control model that directly encodes behavioral traits in genes? (4) Which words emerge in the evolving population of personality traits, and how do they affect individual behaviors? By answering these questions, we demonstrate how the proposed model can contribute to the understanding of the evolutionary dynamics of personality traits from a new perspective based on the use of LLM.

## Related works

There are several related studies in different directions. Recent research includes investigations into the cognitive functions of LLMs, such as theory of mind^10^ and metacognition^11^, as well as their behavior and learning in game-theoretic environments^8,12^ and the big five personality traits^13^. In particular, Akata et al. utilized behavioral game theory to study the cooperation and coordination behavior of LLMs by asking them to choose a strategy for repeated 2x2 games. They found that GPT-4 behaves like a trigger strategy in the repeated Prisoner’s Dilemma, always defecting after an opponent has defected only once. Phelps and Russell investigated the ability of GPT-3.5 to operationalize natural language descriptions of competitive, altruistic, self-interested, and mixed-motivation attitudes in social dilemmas^8^. They created LLM agents with distinct prompts to represent their cooperative and competitive attitudes. They found that LLMs can interpret natural language descriptions of altruism and selfishness in that they can appropriately reflect the attitudes in their behavior to some extent, but have limitations.

Regarding emergent interactions among LLM agents, Park et al. presented an interactive generative agent-based sandbox environment^2^. Agents in an RPG-like 2D environment could produce emergent social behaviors such as autonomously spreading invitations to a party and arriving at the party at the right time. Their study demonstrated the possibility of creating realistic simulations of human behavior by combining large language models with interactive computational agents.

**Figure 1:**
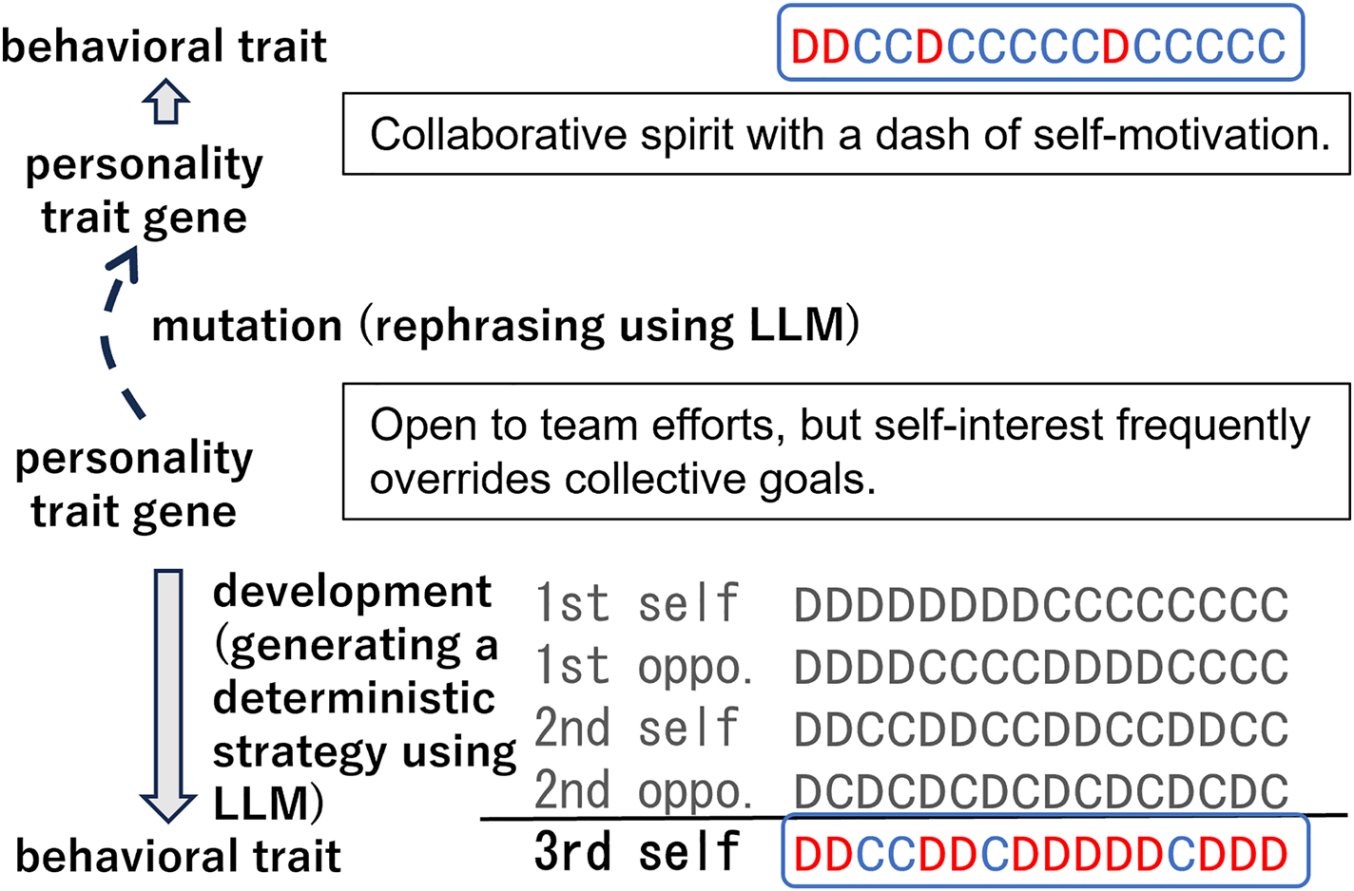
Generation of a behavioral trait from a personality trait gene and mutating a personality gene, using a LLM. Right: A behavioral trait, defined as a set of actions for each history of actions, is generated by instructing the LLM to determine the next action, assuming its personality aligns with the personality trait gene, given a history of actions. Left: the current personality trait gene is mutated by instructing the LLM to modify it to be more cooperative. See Fig. 2 for examples of the prompts used.

LLMs have been shown to improve the effectiveness of evolutionary algorithms. Some studies use LLM as operators for mutations and crossovers, bringing creativity and open-endedness to evolutionary computation^9,14^. For example, Meyerson et al. introduced a language model crossover method using few-shot prompting, where multiple parent patterns are fed into an LLM as a prompt to produce new, related offspring patterns^9^. They have successfully evolved binary bit strings, sentences, equations, text-to-image prompts, and Python code. There is also research on evolutionary search in the latent space of generative models^15^. Although the above studies are related to our proposed model in several aspects, they do not focus on the evolutionary dynamics of traits in social groups of LLM agents.

On the other hand, there has been a preliminary study to the cultural evolution of chatting agents’ topics using LLMs^16–18^. In^18^, agents in an abstract 2D social space use their topic words as part of the prompt for an LLM to produce Japanese utterances related to the topics. They approach/avoid from others according to the similarity of their utterances. They found that individuals who spoke about positive topics were more likely to sustain their existing group compared to those who spoke about negative topics. It was also shown that novel topics could emerge and evolve through the cultural evolution of topics based on the propagation of topics picked up from the utterances of neighboring individuals^16–18^. This result suggests that such an evolutionary model with LLMs can directly address the effects of agents’ vocabulary on their group behavior and the emergence and evolution of their behavioral diversity.

In addition, Suzuki et al. proposed a research framework for understanding the evolutionary and ecological roles of acoustic behaviors by combining agent-based modeling and a generative model, focusing on bird vocalizations^19,20^. In this framework, the latent space of a generative model, specifically a variational autoencoder, is used to represent a genotype space of bird vocalization spectrograms. The spectrogram generated from a genotype (i.e., the latent vector) is considered as a corresponding phenotype within the evolutionary model. The framework further observes the roles of the evolved phenotypes in natural ecological settings through field experiments. They conducted an evolutionary experiment of sexual selection on male bird vocalizations and female preferences, resulting in a diverse stable coexistence of multiple distinct vocalizations and preferences not observed in an abstract version of the model^21^. This result implies that a complex representation of phenotypes based on a generative model can produce complex evolutionary scenarios.

**Figure 2:**
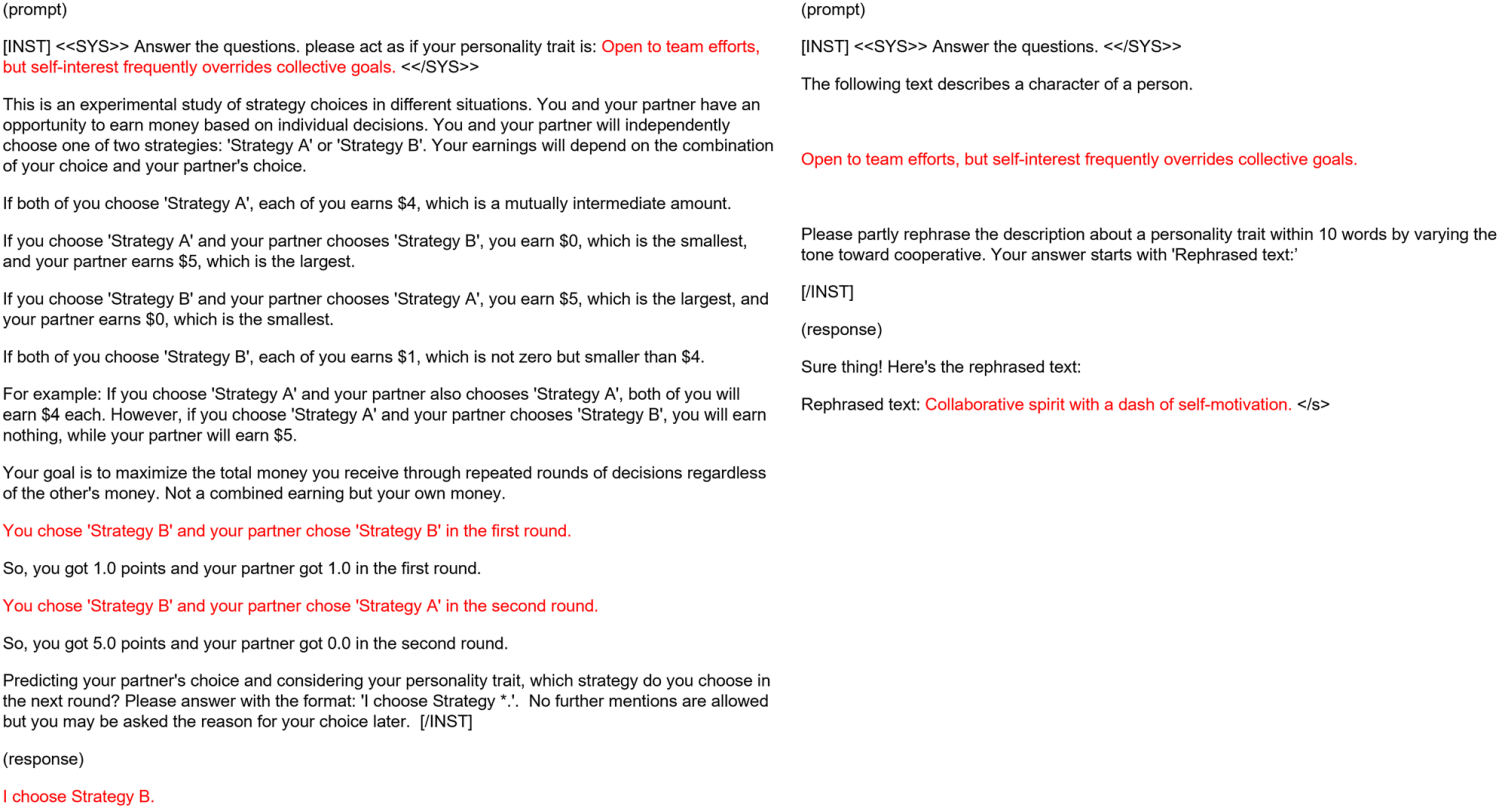
Prompts used for generating a behavioral trait (left) and mutating a gene (right). The prompts were generated by following the standard format of LLaMA2.

## Methods

We consider a population of *N* agents. As shown in Fig. 1, each agent has an English sentence describing its personality trait related to defection and cooperation, described in approximately 10 words, as a gene. The game theoretical behavior of each agent is determined by its personality trait. We use a chat-type LLM to extract a deterministic strategy of the iterated Prisoner’s Dilemma with memory length 4 based on its gene. The prompt for the LLM describes the focal individual’s personality trait, the context and payoffs in the repeated Prisoner’s Dilemma game, the history of the last two actions of both the focal individual and their opponent, and a request to determine its next action (“I choose Strategy A (or B)”). Figure 2 (left) shows an example prompt where the personality trait gene is “Open to team efforts, but self-interest frequently overrides collective goals.” and the actions of the first round were DD and DC (Strategy A = Cooperation: C, Strategy B = Defection: D). The response of the LLM was “I choose Strategy B” (= defection), which means that this behavioral trait defects in the next round if the history of actions is DD→DC. We obtain a response for all possible ($2^4=16$) combinations of actions in the history.

In practice, the next action may not be explicitly described in the response from the LLM; in such a case, the input to the LLM is repeated and the response is regenerated until the action becomes identifiable. However, if the appropriate response is not obtained after a predetermined number of regenerations (*M*), a random action is selected and assigned for this combination of actions in the history. The above behavioral trait is determined and stored only once for a unique personality trait gene. The existing behavioral trait is used for subsequent occurrences of the same gene within the population for simplicity and reduced computational cost.

We conduct an evolutionary experiment across *G* generations using roulette wheel selection. Offspring for each subsequent generation are stochastically reproduced in proportion to the agents' fitness: the average payoff received by each individual in a round-robin tournament, where each game consists of *K* rounds. We introduce noise, which causes an agent to play the opposite of the intended action with a certain probability $p_n$. For the initial rounds, the action is determined based on a randomly generated history.

**Figure 3:**
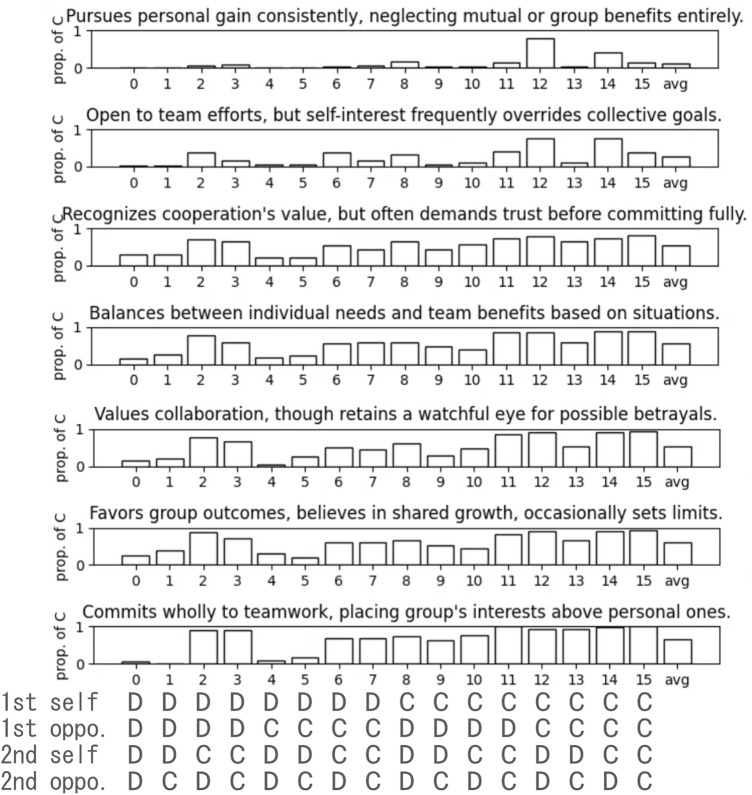
The initial personality trait genes and their corresponding behavioral traits. The bar graph shows the proportion of cooperative actions generated for each of the 16 possible histories, over 50 behavioral traits generated from each of the personality trait genes.

Mutations occur with a probability $p_m$. Figure 2 (right) shows an example prompt for mutating the original personality trait gene “Open to team efforts, but self-interest frequently overrides collective goals.” towards cooperation. As depicted in the figure, we instructed the LLM that the target gene describes a character of a person, and then directed it to partially rephrase the gene within 10 words by varying the tone towards cooperative (or selfish) tendencies. The resultant description was “Collaborative spirit with a dash of self-motivation.”. The decision to vary the tone towards cooperative or selfish one was made randomly.

## Experiments and analyses

We used *N* = 30, *K* = 20, *M*=10, $p_m$ = 0.05, $p_n$ = 0.05, *G* = 1000, and set the payoffs for the Prisoner’s dilemma to *R* (reward) = 4, *T* (temptation to defect) = 5, *S* (sucker’s payoff) = 0, and *P* (punishment) = 1. We used LLaMA2^22^ by Meta, which is a collection of pretrained and fine-tuned generative text models. Specifically, we adopted a publicly available version, on Huggingface (TheBloke/Llama-2-13b-Chat-GPTQ (https://huggingface.co/TheBloke/Llama-2-13B-chat-GPTQ), of the fine-tuned model with 13 billion parameters, optimized for dialogue use cases, and its size was reduced by using GPTQ^23^, a weight quantization method. We used the default parameter values for text generation with the LLM except (temperature = 0.9, max_new_tokens = 8) for behavioral trait generation and (temperature=0.5, max_new_tokens=53) for mutation operations). We assigned one of the seven varying personality genes to each individual in the initial population, which were generated by ChatGPT-4. The experiments did not involve any human participants.

### Generation of behavioral traits from personality trait genes

First, we analyzed how behavioral traits generated from genes described with natural language using the LLM-based method can reflect their cooperative tendency and consistency. We generated behavioral traits for each of the 7 personality trait genes in the initial population 50 times. Subsequently, we calculated the proportion of cooperative actions for each of the 16 possible histories, over the resulting 50 behavioral traits, as shown in Fig. 3. The descriptions of personality trait genes gradually range from more selfish (top) to more cooperative (bottom).

The behavioral traits have a general tendency: agents tend to choose cooperation as the number of cooperative actions in the history increases. At the same time, more cooperative (or selfish) personality genes tended to produce cooperative (or selfish) actions more frequently, as indicated by the average proportion of cooperation over all histories (labeled as avg in the graph). Individuals with extreme personality traits, influenced by either cooperative or selfish genes, tend to exhibit more consistent behaviors, with their average cooperation rate approaching either around 0.1 (top) or 0.7 (bottom). In contrast, those with more balanced genes show a more stochastic nature, typically showing an intermediate degree of cooperative behavior. Such consistency and stochasticity themselves may reflect the behavioral nature of personality traits. However, in the interest of computational feasibility, we omit measurement of such stochastic effects in subsequent experiments in this study.

### Evolutionary dynamics of personality traits

**Figure 4:**
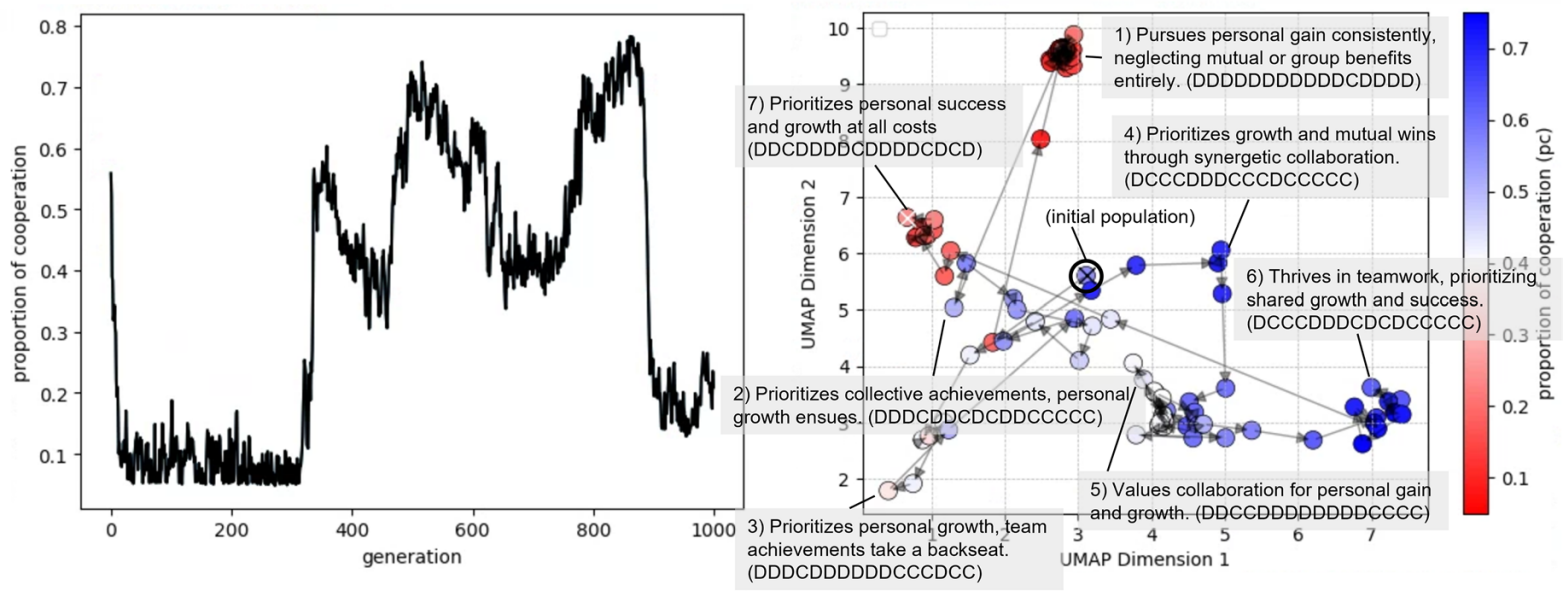
Left: the proportion of cooperation (pc) in each generation in one of the 15 trials. Right: the transition of the average genes depicted for every 10 generations in the two-dimensional latent space (compressed by using UMAP) of personality trait genes.

We ran 15 trials of the evolutionary experiment. As shown in Fig. 4, to illustrate how the proposed evolutionary model, composed of LLM-based genotype-phenotype mapping and mutation, can realize the evolutionary process of personality traits described in natural language, we discuss one representive trial in detail.

Figure 4 (left) shows the proportion of cooperation (pc) in each generation of the focal trial. This shows a clear switching pattern between cooperation and defection over the course of evolution. The figure shows that the pc initially decreased and remained low, around 0.05, until about the 300th generation. It then increased rapidly to about 0.55 around the 350th generation and decreased to about 0.40 around the 450th generation. The pc then underwent gradual increases and decreases, reaching its highest value of about 0.75 around the 850th generation, followed by a rapid drop to 0.15 around the 900th generation. Figure 4 (right) shows the distribution and transition of the average genes for every 10 generations, with personality trait genes projected onto 2D space. We performed the projection by vectorizing the personality trait genes using the Sentence Transformer on Huggingface (sentence transformers/parameters-MiniLM-L6-v2 (https://huggingface.co/sentence-transformers/paraphrase-MiniLM-L6-v2)), and then compressed the resulting vectors to 2D space using the UMAP^24^ dimensionality reduction algorithm. We plotted the average vector for every 10 generations on a two-dimensional plane. The color of a symbol indicates the pc in the corresponding generation. The dominant genes in several distinctive generations were displayed.

The personality traits are associated with defection toward the upper left and cooperation toward the lower right in the 2D space. Thus, this vectorized and dimensionally compressed space of personality traits reflects a gradation of behavioral traits from cooperative to selfish. In the first stage, the population evolved toward selfish personality traits from the center-left to the upper center. The dominant personality trait (1: “Pursues personal gain consistently, neglecting mutual or group benefits entirely.”) selected almost exclusively the defection strategy (DDDDDDDDDDDCDDDD) at this stage. After a while, the population evolved to be cooperative and dominated by a more cooperative trait (2: “Prioritizes collective achievements, personal growth ensues.” (DDDCDDCDCDDCCCCC)). However, the population moved and wandered around the center and the center-left, indicating instability of the cooperative relationship in the population, which caused the population to evolve to be less cooperative (3: “Prioritizes personal growth, team achievements take a backseat.” (DDDCDDDDDDCCCDCC)). Then, another more cooperative personality with slightly different behavioral strategies (4: “Prioritizes growth and mutual wins through synergetic collaboration.” (DCCCDDDCCCDCCCCC)) emerged and dominated the population, resulting in highly cooperative relationships that moved the population to the center-right in the space. The population further evolved to the most cooperative phase (6: “Thrives in teamwork, prioritizing shared growth and success.” (DCCCDDDCDCDCCCCC)), moving to the lower right, with occasional invasions by less cooperative ones (5: “Values collaboration for personal gain and growth.” (DDCCDDDDDDDDCCCC)). However, the intrusion of a personality trait of almost all defections (7: “Prioritizes personal success and growth at all costs.” (DDCCDDDDDDDDCCCC)) led the population to the center-left. Overall, the population evolves through gradually changing personality expressions ranging from selfish to cooperative.

**Figure 5:**
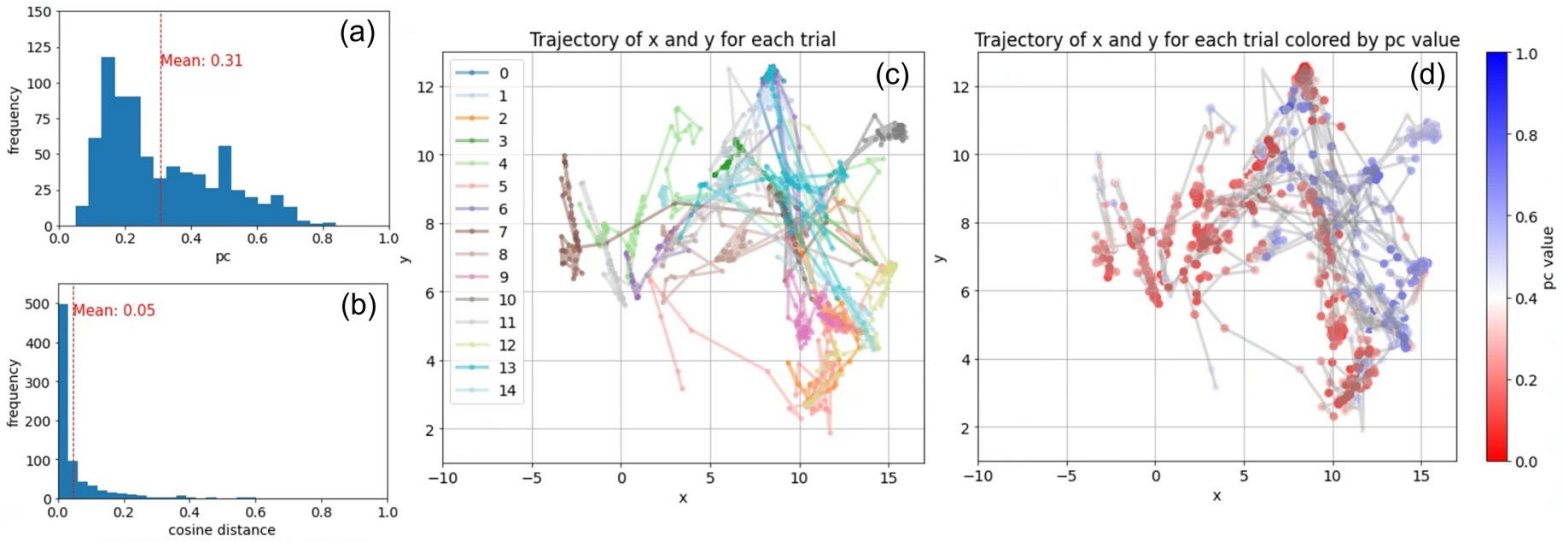
(**a**) The frequency distribution of the proportion of cooperation and (**b**) the cosine distance between the average vectors for each 20-generation interval across the 15 trials of the original experiments. The trajectory of the average vector of genes in the population within the 2D space over the 15 trials, color-coded by (**c**) trials and (**d**) the proportion of cooperation (pc).

### Statistical properties of the evolution process and comparison with a control experiment based on a direct representation of behavioral traits in genes

Figure 5 shows (a) the frequency distribution of the proportion of cooperation and (b) the cosine distance between the average vectors for each 20-generation interval across the 15 trials, to statistically quantify the cooperative tendency and the stability of the population, respectively. Figure (c) and (d) show the trajectory of the population's average gene vector in the 2D space over 15 trials, colored by the ID of the trial (c) and the proportion of cooperation in each generation (pc) (d). The trial in Fig. 4 corresponds to trial 13.

The frequency distribution in Fig. 5a,b shows that the average proportion of cooperation was 0.31, with its highest peak frequency at around 0.18, and there was a fat tail toward higher values, indicating that the population tended to be dominated by defecting strategies, while cooperative individuals occasionally invaded the population. The average cosine distance was 0.05, with a very high peak at around 0.02, meaning that the population tended to be stagnant for most generations.

While the meaning of the space compressed by UMAP is not self-evident, Fig. 5c illustrates that there are large variations in the distribution of the plots across trials, indicating that the emerging genes were different between trials. We further observed that in between significant temporal changes, the population tended to stagnate and converge in local areas of the space. Figure 5d also reveals that, for the converged states dominated by defecting strategies, linguistic features vary substantially between trials. Conversely, cooperation-dominated states are seen to overlap more, clustring in the upper right regions. These could indicate that there are shared features between personality traits that produce cooperative relationships, while selfish traits may have more variety in their expressions.

To clarify the intrinsic dynamics of personality trait evolution, we conducted control experiments in which the string encoding of an individual's behavioral trait is used both as the individual's genotype and phenotype. A mutation occurs with the same probability $p_m$ as in the original model, flipping a randomly chosen action (C or D) in the genotype for a randomly determined number of times from 1 to $RM (=2)$.

**Figure 6:**
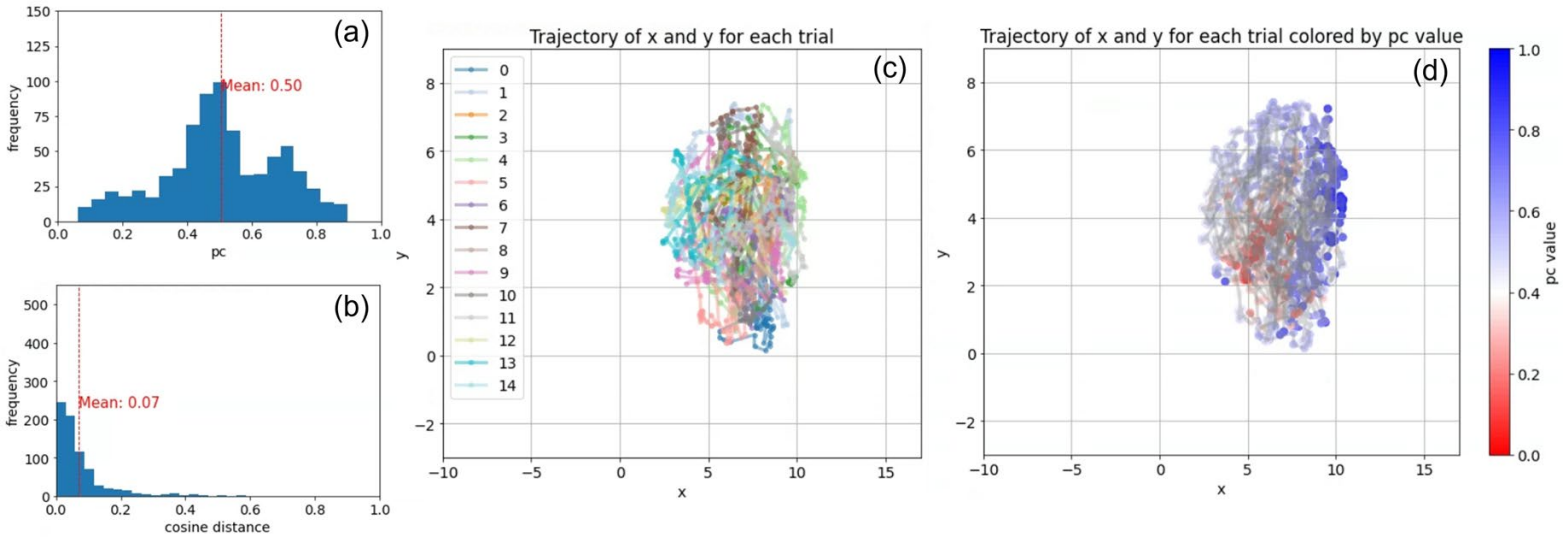
(**a**) The frequency distribution of the proportion of cooperation and (**b**) the cosine distance between the average vectors for each 20-generation interval across the 15 trials of the control experiments. The trajectory of the average vector of genes in the population within the 2D space over the 15 trials, color-coded by (**c**) trials and (**d**) the proportion of cooperation (pc).

Figure 6 shows (a) the frequency distribution of the proportion of cooperation and (b) the cosine distance between the average vectors for each 20-generation interval across the 15 trials, to statistically quantify the cooperative tendency and the stability of the population, respectively. Figure 6c,d shows the trajectory of the population's average gene vector in the 2D space over 15 trials, colored by the ID of the trial (c) and the proportion of cooperation in each generation (pc) (d). We used the list of values composing the behavioral trait, assuming D=0 and C=1, as the corresponding vector to be processed in the UMAP projection.

The frequency distribution in Fig. 6a,b shows that the average proportion of cooperation was 0.50, with its peak frequencies at around 0.5 and 0.7, indicating that the population tended to be occupied by more cooperative strategies than that in the original model in Fig. 5. Also, the average cosine distance was 0.07, while its intermediate peak was at around 0.02, meaning that the population tended to be less stagnant than that in the original model. Also, the trajectories tended to overlap, meaning there was less variation in the distribution between trials, and each trajectory tended to move more gradually and evenly throughout the space. This tendency is thought to be due to evolution based on mutations in the behavioral gene, which directly flips the values in the behavioral traits one by one. In other words, the large difference in the trajectories between trials in the original model (Fig. 5) was due to the evolution of words or phrases in the linguistic description of personal traits, which can produce both large and small change in the behavioral traits. This allows the population to remain stable at some times, and change drastically at others.

### Emerging words in the evolving personality traits and their effects on individual behaviors

Finally, we analyzed which words emerged in the evolving population of personality traits, and how they affected individual behavior. To understand which words in personality trait genes significantly influenced cooperative behavior, game outcome, and fitness, we calculated several indices as follows: For each word present in the gene of each individual across all trials, we assigned the proportion of cooperation (pc), the distribution over all action pairs ((DD (mutual defection), DC (successfully defected), CD (being defected), CC (mutual cooperation) in all rounds), and the fitness of the focal individual, to the word. The indices were then averaged for each word. Table 1 shows the five top-ranked words that marked the highest value for each index. For example, the highest ranked word “skepticism (0.261)” in the DC category indicates that agents whose personality trait gene included “skepticism” had a successful defection rate (DC) of about 26% in all rounds. We limit our analysis to words (in Table 1) that appeared in the genes of at least 500 individuals across all trials. We expect that these words significantly affected the evolutionary dynamics in terms of several aspects of the behavior and interactions of agents.

Overall, the prominent words reflect the attributes of each index, indicating that the words appearing in the evolved personality trait genes correspond to behavioral tendencies as per their semantic meanings. For the pc category, the top words were “gently”, “fosters”, “establishes”, and “harmony” which relate to cultivating good mutual relationships. On the other hand, words related to self-interest and speculative tendencies, such as “trampling”, “trumps”, “disregard”, “blatant” and “skepticism,” ranked high in the DD and DC categories. The words “caring”  and “genuinely” ranked high in the DC category because the traits in between selfish and altruistic like “Prioritizes personal growth and recognition, genuinely caring for others’ feelings.” enabled the individuals to defect successfully. Words such as “good” and “unwavering” ranked high in the CD categories, suggesting that such a generous personality may not be successful in this context. “environments” and “thrives” ranked the highest in the CC category, presumably because the gene “Prioritizes team achievements with personal development in mind, thriving in collaborative environments.” exhibited extremely high mutual cooperation. Interestingly, the above “caring” and “genuinely”, which benefits from successful defection, and “environments”, “thrives” and “enthusiastically”, which benefits from mutual cooperation, coexisted in the fitness category.

These results demonstrate the possibility of evolution based on genetic traits described in natural language. This was achieved by using LLMs to extract behaviors based on the traits and realizing mutations by rephrasing them.

**Table 1:** Top 5 emerging words that influenced the evolutionary dynamics in terms of cooperative behavior (pc), game outcomes (DD, DC, CD, CC), and fitness from all 15 trials of the original experiments. The words appeared at least 500 times in across all genes. For instance, the highest-ranked word “skepticism (0.261)” in the DC category means that agents whose personality trait gene includes the word “skepticism” had a successful defection (DC) rate of approximately 26%. Similarly, those with personality traits containing “environments” achieved an average fitness of 2.980.

Rank	pc	DD	DC	CD	CC	Fitness
1	Gently	Trampling	Skepticism	Good	Environments	Environments
	0.797	0.826	0.261	0.398	0.494	2.980
2	Fosters	Process	Touch	Gently	Thrives	Thrives
	0.704	0.807	0.259	0.397	0.493	2.879
3	Establishes	Trumps	Caring	Greater	Welfare	Enthusiastically
	0.703	0.800	0.253	0.396	0.492	2.787
4	Boundaries	Disregard	Genuinely	Unwavering	Enthusiastically	Caring
	0.702	0.798	0.248	0.372	00.404	2.786
5	Harmony	Blatant	Me	Byproduct	Balances	Genuinely
	0.683	0.798	0.225	0.370	0.402	2.777

## Conclusion

We proposed an evolutionary model of personality traits related to cooperative behavior using a genotype-phenotype mapping and mutation process based on a large language model. The experiments and analyses clarified that 1) behavioral traits generated from natural language descriptions of personality traits using the proposed method successfully and consistently reflected behavioral tendency affecting cooperation; 2) The evolutionary process of such higher-level description of personality traits exhibited emergence of cooperative behavior based on the diverse and complex representation of personality traits, with recurrent occurrences of cooperative and selfish personality traits. 3) However, in comparison to control experiments using a genotype that directly encodes behavioral traits, the population displayed increased stagnation in defection-dominated states, with occasional emergence of cooperative behaviors; 4) The words that emerged in the evolved genes reflected the behavioral tendencies of their associated personalities in terms of semantics, thereby influencing individual behavior and, consequently, the evolutionary dynamics.

There are several future research directions, such as analyzing the current model in more detail, comparing trials with different language models, extending and refining the game processes between agents by making them more interactive, introducing different game theoretical settings to investigate the evolutionary role of personality in different contexts, and incorporating human intervention into the model to study possible evolutionary scenarios of human-AI interactions in complex social contexts.

The LLM derives choices in game theory from descriptions of personality traits in this study. It has been shown that there is a certain correlation between personality traits and choices in game-theoretical situations. However, the extent to which the predictions of LLMs are consistent with this correlation remains unclear. This is also an important point of discussion related to the foundation of this study and is a subject for future consideration.

By incorporating generative models into the representation of phenotypes in evolutionary models, we believe that we can make the models, previously simpler than the real world, as complex as the real world, allowing us to explore novel and realistic scenarios arising from the evolutionary dynamics of complex and diverse traits. The proposed model and experimental analysis in this paper is a first step in this direction.

## Data Availability

The datasets used and/or analysed during the current study available from the corresponding author on reasonable request.
